# C3a Increases VEGF and Decreases PEDF mRNA Levels in Human Retinal Pigment Epithelial Cells

**DOI:** 10.1155/2016/6958752

**Published:** 2016-09-22

**Authors:** Qin Long, Xiaoguang Cao, Ailing Bian, Ying Li

**Affiliations:** ^1^Department of Ophthalmology, Peking Union Medical College Hospital, Chinese Academy of Medical Sciences and Peking Union Medical College, Beijing, China; ^2^Key Laboratory of Myopia, Ministry of Health, Fudan University, Shanghai, China; ^3^Department of Ophthalmology, People's Hospital of Peking University, Beijing, China

## Abstract

Complement activation, specifically complement 3 (C3) activation and C3a generation, contributes to an imbalance between angiogenic stimulation by vascular endothelial growth factor (VEGF) and angiogenic inhibition by pigment epithelial derived factor (PEDF), leading to pathological angiogenesis. This study aimed to investigate the effects of C3a and small interfering RNA (siRNA) targeting C3 on the levels of VEGF and PEDF mRNAs in human retinal pigment epithelial (RPE) cells. ARPE-19 cells were cultured in the presence of exogenous C3a at 0.1 *μ*M and 0.3 *μ*M C3a for 24, 48, and 72 hours. 0.1 pmol/*μ*L duplexes of siRNA targeting C3 were applied for C3a inhibition by transfecting ARPE-19 cells for 48 hours. RT-PCR was performed to examine the level of VEGF and PEDF mRNA. A random siRNA duplex was set for control siRNA. Results demonstrated that exogenous C3a significantly upregulated VEGF and downregulated PEDF mRNA levels in cultured ARPE-19 cells, and siRNA targeting C3 transfection reversed the above changes, significantly reducing VEGF and enhancing PEDF mRNAs level in ARPE-19 cells compared to the control. The present data provided evidence that reducing C3 activation can decreases VEGF and increase PEDF mRNA level in RPE and may serve as a potential therapy in pathological angiogenesis.

## 1. Introduction

Choroidal neovascularization (CNV) is one of the leading causes of irreversible central blindness in the various ocular disorders, such as age related macular degeneration (AMD), pathologic myopia (PM), and polypoidal choroidal vasculopathy (PCV) [1–3]. Choroidal angiogenesis is a highly complex biological process that involves a delicate balance between angiogenic and antiangiogenic factors, each regulated by multiple control systems [4, 5]. Among the cytokines involved in pathological angiogenesis, vascular endothelial growth factor (VEGF) and pigment epithelial derived factor (PEDF) serve as the most potent angiogenic stimulators and angiogenic inhibitors, respectively [1, 6].

Recently, the role of the complement system on the development of choroidal pathological angiogenesis has received considerable attention [7, 8]. The complement system is part of the innate immune system and is composed of approximately 30 structural proteins and receptors that promote or inhibit complement activation [9]. Complement 3 (C3) is the key component in the complement cascade with the cleavage of C3 and the formation of C3a marking the activation of complement system [10]. Evidence has shown elevated complement 3a (C3a) levels in plasma and local tissue of choroidal pathological angiogenesis compared to normal controls [11, 12]. Moreover, our recent study suggested that serum C3 levels may be a predictive risk factor for CNV formation in PM patients, suggesting interplay between innate immunity and abnormal choroidal angiogenesis [13]. Retinal pigment epithelial (RPE) cells are a major source of choroidal angioregulatory proteins and play an important role in maintaining the angiogenic homeostasis through counterbalancing VEGF angiogenic stimulation and PEDF inhibition [14]. Recent studies that focused on complement activation at the level of the RPE indicated that these cells can express various complement receptors, including the C3a receptor (C3aR) [15]. In addition, genetic ablation of C3a receptors reduces VEGF expression and CNV formation in vivo, and pharmacological blockade of C3aR also reduces CNV [12]. However, the underlying effect of C3a on the level of VEGF and PEDF in RPE has not yet been clarified.

The aims of this study were twofold: (1) to investigate the effect of C3a on the level of VEGF and PEDF in cultured RPE cells through treatment with exogenous C3a and (2) to investigate the effect of attenuating C3a on VEGF and PEDF mRNA levels in cultured RPE cells achieved through silencing C3 using small interfering RNA (siRNA), which is one of the most efficient methods for selectively knocking down gene expression. Our hypothesis was that, considering C3a as an injury factor for pathological angiogenesis by enhancing the local VEGF level in RPE, siRNA targeting C3 may serve as a protective factor for the regulation of pathological angiogenesis including CNV by rescuing VEGF and PEDF mRNA level changes caused by C3a stimulation in the RPE layer.

## 2. Materials and Methods

### 2.1. RPE Cell Line Culture

ARPE-19 cells, a human RPE cell line obtained from the Eye Laboratory of Peking University People's Hospital, were cultured in 10 cm dishes. Briefly, ARPE-19 cells were maintained in Dulbecco's modified Eagle's medium-Ham's F12 1 : 1 (DMEM/F12) supplemented with 15% fetal bovine serum (FBS) with 100 *μ*g/mL penicillin and streptomycin at 37°C in a humidified environment containing 5% CO_2_. The ARPE-19 cell line was passaged twice weekly to maintain cell confluence around 70%. All reagents used for cell culture were purchased from Gibco by Invitrogen (Carlsbad, CA, USA) unless otherwise indicated in the text.

### 2.2. C3a Treatment

For human C3a treatment, ARPE-19 cells (4 × 10^5^) were seeded in six-well plates and, 24 hours later, the cells were incubated with fresh medium for an additional 24 hours and then starved in serum-free MEM for 24 hours. The cells were then treated for 24, 48, and 72 hours with 0.1 or 0.3 *μ*M recombinant human C3a (Cat 204881; Millipore, Billerica, MA, USA).

### 2.3. Application of Short Interfering RNA (siRNA)

A duplex siRNA was designed to target human C3 (NCBI: NM_000064) and was purchased from Origene (SR300499; Rockville, MD, USA). A nonsilencing siRNA duplex targeting sequence, AATTCTCCGAACGTGTCACGT, was used as control siRNA (random siRNA duplex). ARPE-19 cells were seeded in six-well plates at a density of 5 × 10^5^ cells per well to obtain 70–80% confluence. Transfection of the siRNA was performed with Lipofectamine® RNAiMAX (Cat 13778075; Life Technologies, Carlsbad, CA, USA) in culture medium (without FBS and antibiotics) according to the manufacturer's instructions. Specifically, 30 pmol siRNA diluted with 150 *μ*L Opti-MEM medium was mixed with diluted transfection reagent (9 *μ*L transfection reagent was diluted with 150 *μ*L Opti-MEM medium) at 1 : 1 ratio. Then the mixture was incubated at room temperature for five minutes and the siRNA-lipid complex was added to the cells. 48 hours after transfection of siRNA (at a concentration at 0.1 pmol/*μ*L), reverse transcription-polymerase chain reaction (RT-PCR) analysis was performed to examine the gene silencing effect. 48 hours after transfection of siRNA targeting C3, cells were stimulated with 0.1 *μ*M C3a (Cat 204881; Millipore) for an additional 24 hours and control cells were transfected with control siRNA.

### 2.4. Semiquantitative RT-PCR Analyses

Total RNA was extracted from cultured ARPE-19 cells with TRIZOL (Life Technologies; Carlsbad, CA, USA) according to the manufacturer's instructions. Aliquots of total RNA were reverse transcribed into single-stranded cDNA by incubation with Moloney murine leukemia virus reverse transcriptase (Cat 6110A; Takara Biochemicals, Kusatsu, Japan). Diluted cDNA products were then subjected to PCR using Ex Taq (Cat RR006A; Takara Biochemicals). The primers used to amplify C3a, VEGF, and PEDF transcripts are listed in Table 1. Human GAPDH RNA was amplified as an internal control. PCR was performed in a DNA thermal cycler (GeneAmp® PCR System 9700, Applied Biosystems; Foster City, CA, USA). Semiquantitative analysis was achieved at a fixed PCR cycle number (32), using a range of total cDNA concentrations within the exponential phase of the PCR. Coamplification of the transcript of interest with internal controls allowed comparison between different RNA samples. PCR products were visualized on agarose gels stained with ethidium bromide and were analyzed using Image J2X software (free software developed by Wayne Rasband, National Institutes of Health [NIH], Bethesda, MD, USA), with level of GAPDH used for normalization.

### 2.5. Statistical Analysis

Data were analyzed using IBM SPSS 19.0 for Windows statistical software (SPSS, Chicago, IL) and GraphPad Prism 5 (GraphPad Software, La Jolla, CA). Data are presented as mean ± SD. The Shapiro-Wilk test was used to test data normality. Statistical analysis used parametric one-way analysis of variance (ANOVA) followed by Tukey Multiple Comparison test and unpaired *t*-test. *P* < 0.05 was considered statistically significant.

## 3. Result

### 3.1. Effects of Exogenous C3a on the Levels of VEGF and PEDF mRNA in Cultured ARPE-19 Cells

**Figure 1:**
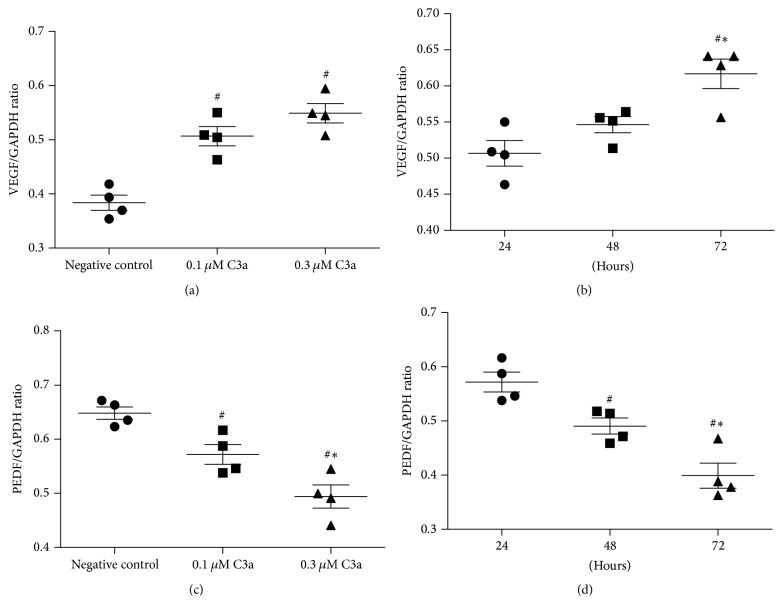
Effect of exogenous C3a on the levels of VEGF and PEDF mRNA in cultured ARPE-19 cells. Total RNA was extracted and mRNA evaluated by RT-PCR analysis. The ratio of the abundance of each mRNA to that of GAPDH was evaluated by densitometric analysis. Data are mean ± SD (*n* = 4). (a) and (c) show the levels of VEGF and PEDF mRNA incubated with different doses of C3a for 24 hours; ^#^ *P* < 0.05 versus negative control cells; ^*∗*^ *P* < 0.05 versus 0.1 *μ*M C3a incubation; (b) and (d) show the levels of VEGF and PEDF mRNA incubated with 0.1 *μ*M C3a for 24, 48, and 72 hours. ^#^ *P* < 0.05 versus 24 hours of incubation; ^*∗*^ *P* < 0.05 versus 48 hours of incubation.

Under treatment with exogenous C3a, the level of VEGF mRNA in ARPE-19 cells significantly increased compared to the negative control (no C3a treatment) (one-way ANOVA; *q* = 7.39 and 9.94, *P* < 0.05); however, there was no statistical significance between 0.1 *μ*M and 0.3 *μ*M C3a treatment (one-way ANOVA; *q* = 2.55, *P* > 0.05) (Figure 1(a)); the level of VEGF mRNA was significantly lower when treated with C3a for 24 and 48 hours compared to 72 hours (one-way ANOVA; *q* = 6.51 and 4.17, *P* < 0.05) (Figure 1(b)); however, there was no significant difference between 24 and 48 hours of treatment (one-way ANOVA; *q* = 2.34, *P* > 0.05). Exogenous C3a significantly decreased PEDF mRNA levels in ARPE-19 cells in a time- and dose-dependent manner (one-way ANOVA; *P* < 0.05) (Figures 1(c) and 1(d)).

### 3.2. Effects of siRNA Targeting C3 on the Level of C3a mRNA in Cultured ARPE-19 Cells

**Figure 2:**
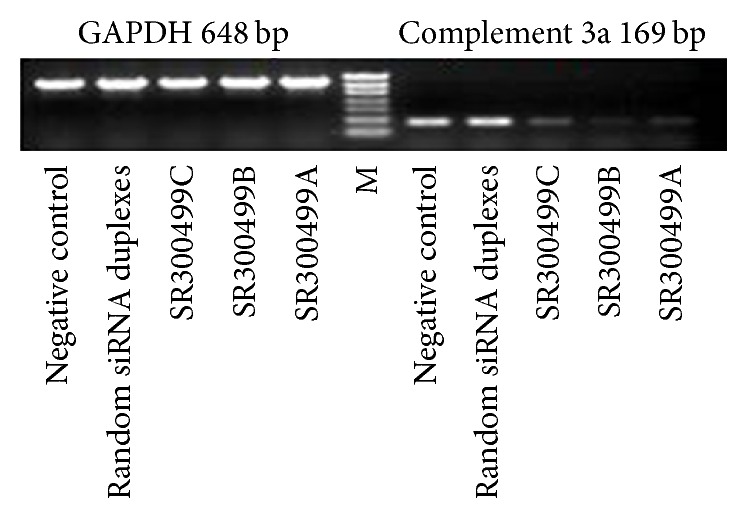
Effect of siRNA against C3 on the level of C3a mRNA in cultured ARPE-19 cells. siRNA duplex targeting C3 (SR300499A and B) reduced the level of C3a mRNA compared to negative control and random siRNA.

Three pairs of siRNA duplexes were provided by Origene and one of these was used to detect the mRNA level of C3a, the activated fragment of C3, in order to evaluate the efficacy of siRNA targeting C3 in cultured ARPE-19 cells. Human GAPDH RNA was amplified as an internal control. The effects of the duplex siRNA specific for C3 on the C3a mRNA levels in ARPE-19 cells are shown in Figure 2. Results indicated that 0.1 pmol/*μ*L siRNA targeting C3 (SR300499A and B) significantly decreased the level of C3a mRNA. The siRNA SR300499A was used in the following experiment.

### 3.3. Contribution of Endogenous C3 to VEGF and PEDF mRNA Levels in Cultured ARPE-19 Cells

**Figure 3:**
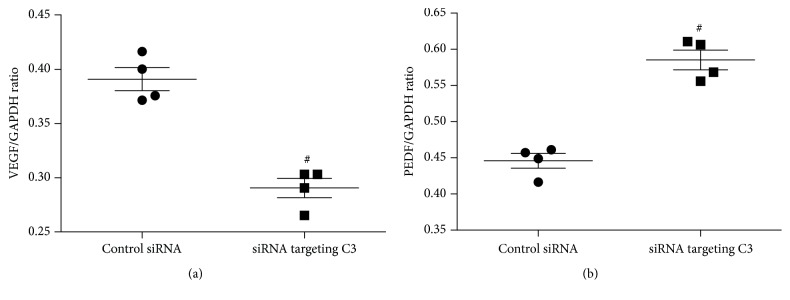
Effect of siRNA targeting C3 on the level of VEGF (a) and PEDF (b) mRNA in cultured ARPE-19 cells. Total RNA was extracted and mRNA evaluated by RT-PCR analysis. The ratio of the abundance of each mRNA to that of GAPDH was evaluated by densitometric analysis. Data are mean ± SD (*n* = 4). (a) The VEGF mRNA was significantly lower in the ARPE-19 cells transfected with 0.1 pmol/*μ*L siRNA targeting C3 for 48 hours compared to control siRNA; (b) the PEDF mRNA was significantly higher in the ARPE-19 cells after transfection of 0.1 pmol/*μ*L siRNA targeting C3 for 48 hours compared to that of transfection with control siRNA. ^#^ *P* < 0.05 versus control siRNA transfected cells.

We used siRNA targeting C3 to evaluate the contribution of endogenous C3 to the VEGF and PEDF mRNA level in cultured ARPE-19 cells, with random siRNA duplex as control siRNA. The results are shown in Figure 3. The level of VEGF mRNA was significantly lower in ARPE-19 cells transfected with 0.1 pmol/*μ*L siRNA targeting C3 for 48 hours compared to control siRNA transfected cells (unpaired *t*-test; *t* = 7.24, *P* < 0.05) (Figure 3(a)). PEDF mRNA levels were significantly higher in ARPE-19 cells after transfection of 0.1 pmol/*μ*L siRNA targeting C3 compared to control siRNA transfection (unpaired *t*-test; *t* = 8.18, *P* < 0.05) (Figure 3(b)).

### 3.4. Effects of siRNA Targeting C3 on the Levels of VEGF and PEDF mRNA in Cultured ARPE-19 Cells Treated with Exogenous C3a

**Figure 4:**
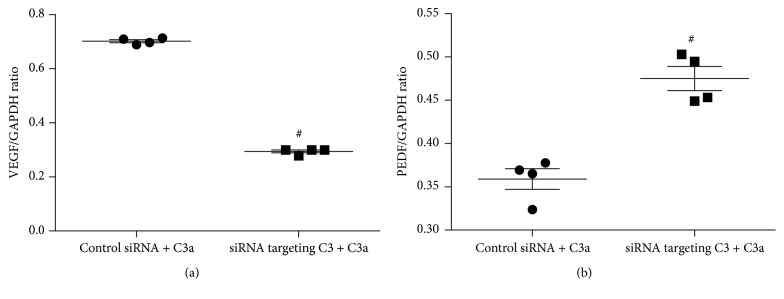
Effect of siRNA targeting C3 on the level of VEGF (a) and PEDF (b) mRNA in cultured ARPE-19 cells treated with exogenous C3a. The ARPE-19 cells were incubated with 0.1 pmol/*μ*L siRNA to C3. Forty-eight hours after transfection of siRNA, cells were incubated with 0.1 *μ*M C3a for an additional 24 hours. The ratio of the abundance of each mRNA to that of GAPDH was evaluated by densitometric analysis. Data are mean ± SD (*n* = 4). (a) VEGF mRNA level was significantly lower in siRNA targeting C3 combined with C3a treated cells compared to control siRNA combined with C3a treated cells; (b) the PEDF mRNA level was significantly higher in siRNA targeting C3 + C3a treated cells compared to siRNA + C3a treated cells; ^#^ *P* < 0.05 versus control siRNA + C3a treated cells.

**Figure 5:**
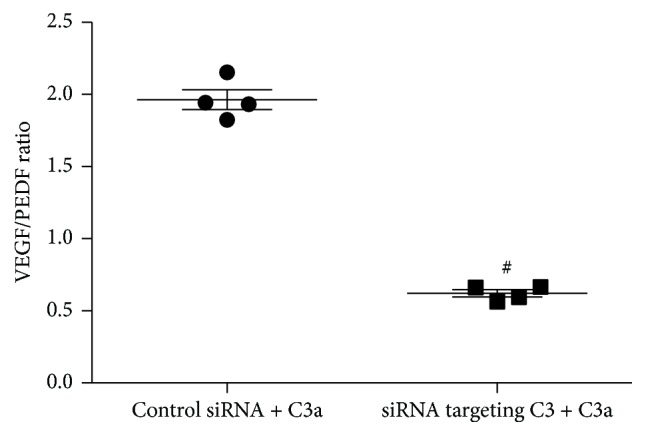
VEGF/PEDF ratio. The ratio of VEGF mRNA/PEDF mRNA in 0.1 pmol/*μ*L siRNA targeting C3 transfected ARPE-19 cells was significantly lower compared to control siRNA transfected cells after exogenous 0.1 *μ*M C3a incubation. ^#^ *P* < 0.05 versus control siRNA + C3a treated cells.

After transfection with 0.1 pmol/*μ*L duplex siRNA targeting C3 or 0.1 pmol/*μ*L duplex control siRNA for 48 hours, ARPE-19 cells were treated with exogenous 0.1 *μ*M C3a for an additional 24 hours with random siRNA duplex set for control siRNA. The levels of VEGF and PEDF mRNA are shown in Figure 4. After exogenous C3a incubation, the ARPE-19 cells transfected with siRNA targeting C3 stimulated significantly lower VEGF mRNA and significantly higher PEDF mRNA compared to control siRNA transfected cells (unpaired *t*-test; *t* = 52.84 and 6.31, *P* < 0.05). The ratio of VEGF mRNA/PEDF mRNA in siRNA targeting C3 transfected cells was significantly lower compared to control siRNA transfected cells (0.62 ± 0.05 versus 1.96 ± 0.14; unpaired *t*-test; *t* = 18.28, *P* < 0.05) (Figure 5).

## 4. Discussion

Our study demonstrated that exogenous C3a increased VEGF and decreased PEDF mRNA levels in cultured human ARPE-19 cells. siRNA targeting C3 inhibited C3a generation reversing the above changes, reducing VEGF but enhancing PEDF mRNAs levels and decreasing the VEGF/PEDF ratio. This supports our initial hypothesis that siRNA targeting C3 may be a potential therapeutic strategy for pathological angiogenesis including CNV by rescuing changes in VEGF and PEDF levels caused by C3a injury.

The RPE is a single layer of epithelial cells with an essential role in maintaining angiogenic homeostasis by secreting a variety of growth factors, expressing multiple receptors and responding to immune stimulators [14, 16]. To date, numerous studies on the pathogenesis of choroidal pathological angiogenesis have focused on the level of RPE and it is recognized as the initial target in multiple proangiogenic signaling [12, 17]. ARPE-19, the human RPE cell line employed in this study, is often used because of its ready availability and stability under prolonged cultivation [18].

VEGF, secreted by the RPE cells, has been identified as one of the critical mediators of angiogenesis, promoting angiogenesis by stimulating proliferation, migration, and tube formation [19]. Current evidence shows that VEGF secretion is increased by RPE cells during choroidal angiogenesis in human and animal models [20, 21]. Moreover, anti-VEGF therapies have exhibited efficacy for angiogenesis inhibition supporting the notion that VEGF plays a key role in pathological angiogenesis including CNV [22, 23].

PEDF is one of the endogenous angiogenic inhibitors secreted in large quantity by native RPE that can downregulate the likely cause of angiogenic activity, VEGF-induced endothelial cell migration and proliferation [24, 25]. Proangiogenic VEGF and antiangiogenic PEDF play counterbalancing roles in the regulation of RPE function [14]. Increasing the VEGF/PEDF ratio in RPE can tip the balance of angiogenic stimulation/inhibition to favor angiogenesis [17]. In contrast, drugs that decrease the VEGF/PEDF ratio may be worth consideration as possible antiangiogenic treatments. Moreover, high levels of PEDF can protect retinal photoreceptors and neurons and thus may promote a favorable clinical outcome [26].

The complement system is composed of three distinct pathways (classical, lectin, and alternative), and recent studies have indicated that inflammation, especially the alternative complement pathway, plays a fundamental role in the pathogenesis of choroidal angiogenesis [27, 28]. C3 and C3a fragments have been reported as important in antiangiogenesis by upregulating RPE VEGF in vitro and in vivo [29]. C3 knockout mice show reduced choroidal vasculogenesis after laser injury due to decreased recruitment of inflammatory cells to the lesion [29]. Additionally, a genetic study has identified a C3 gene associated with CNV in AMD patients [30]. Nozaki et al. detected that C3a upregulated the secretion of VEGF by primary human RPE cells [12]; however, this study also reported that C3a treatment did not increase VEGF secretion by ARPE-19 cells [31].

In the present study we confirmed that exogenous C3a triggered an increase in VEGF mRNA in cultured ARPE-19 cells. In contrast, C3a decreased PEDF mRNA levels in a time- and dose-dependent manner. These results indicated that exogenous C3a may serve as an injury mediator by activating the complement cascade that, in turn, drives the RPE towards pathological angiogenesis.

Inhibition of complement activation with siRNA targeting C3 resulted in downregulation of VEGF mRNA and upregulation of PEDF mRNA leading to the significant reduction of VEGF/PEDF ratio in exogenous C3a treated ARPE-19 cells. Given that siRNA targeting C3 could not influence the effect of exogenous C3a, the difference of VEGF and PEDF mRNA levels between siRNA targeting C3 transfected ARPE-19 cells and the control siRNA transfected cells may be due to the abolished effect of endogenous C3a by C3 silencing. Evidence indicates that an enhanced VEGF/PEDF ratio has the ability to generate a proangiogenic environment while a decreased VEGF/PEDF ratio may favor an antiangiogenic state pertinent to various diseases, including CNV [17, 32]. In our study, we observed a significantly lower VEGF/PEDF ratio (decreased by 68%) in siRNA targeted C3 transfected ARPE-19 cells compared to the control siRNA transfected cells. This alteration in the ratio not only confirmed the proangiogenic role of C3a exposure but also suggests that reduction of C3 could be a potential antiangiogenic treatment. However, there are concerns about C3 as a target for therapy. Firstly, a constant low level of complement activation in the eye serves as a primary defense mechanism against pathogens, and the activation of C3 is a key process in this complement cascade. However, blocking C3 activation may completely block complement activation that in turn may lead to the inhibition of innate immune system. Secondly, our study demonstrated that there is low level secretion of VEGF in the RPE that is in accordance with our previous results and other studies [17]. Although VEGF promotes pathological angiogenesis, it also plays a crucial role in cellular homeostasis and serves as a neurotrophic and neuroprotective factor [33, 34]. Suppression of VEGF by siRNA targeting C3 may exhibit side effects including cell death and vision loss as demonstrated in animal models, cell culture, and human randomized clinical trials after indiscriminate or prolonged VEGF inhibition [35–37]. Therefore, balancing the angiogenic and neuroprotective effect of VEGF is of vital importance in future studies. Thirdly, we observed the upregulation of VEGF and downregulation of PEDF in cultured ARPE-19 cells when treated with exogenous C3a, thus suggesting C3a to be a factor triggering angiogenesis in vivo. Although it has been demonstrated that C3a is generated early in the course of laser-induced CNV [12], the hypothesis that exogenous application of C3a recreates disease conditions needs further verification. Fourthly, although ARPE-19 cells retain many of the characteristics of native RPE cells, including cell morphology and functional tight junctions, and are routinely used as an alternative to primary cultures [18], ARPE-19 cells may include potential phenotypic changes after multiple passages [14, 38, 39]. Therefore, our results still need to be carefully interpreted since ARPE-19 cells may represent only a subset of primary RPE cells.

## 5. Conclusions

In summary, the present study confirmed that C3a increases VEGF but decreases PEDF mRNA levels in ARPE-19 cells and, for the first time, provided evidence that siRNA targeting C3 downregulated the levels of VEGF mRNA and upregulated the levels of PEDF mRNA and thus reduced the VEGF/PEDF ratio in cultured ARPE-19 cells. Our results provide an additional insight into the regulation of pathological angiogenesis and indicate that reducing C3 activation and C3a generation may serve as a potential antiangiogenic therapy for clinical CNV treatment. However, as we mentioned above, balancing the effects of C3 inhibition will be the goal for the treatment of pathological angiogenesis including CNV.

## Figures and Tables

**Table 1:** PCR primers used in this study.

Gene	Forward primer (5′-3′)	Reverse primer (5′-3′)
C3a	GCTGAAGCACCTCATTGTGA	CTGGGTGTACCCCTTCTTGA

VEGF	TGCATTCACATTTGTTGTGCTGTAG	GCAGATTATGCGGATCAAACC

PEDF	GCCTCACCTCCGAGTTCATT	CCGGTGTTCCACCTGAGTC

GAPDH	TCACCATCTTCCAGGAGCGAG	TGTCGCTGTTGAAGTCAGAG
